# Supplementary material for: Chimeric symbionts expressing a Wolbachia protein stimulate mosquito immunity and inhibit filarial parasite development
Source: Commun Biol. 2020 Mar 6;3:105. doi: 10.1038/s42003-020-0835-2 (PMC7060271; doi:10.1038/s42003-020-0835-2)
Supplement: Supplementary file 1 — Supplementary Information [file 42003_2020_835_MOESM1_ESM.pdf]

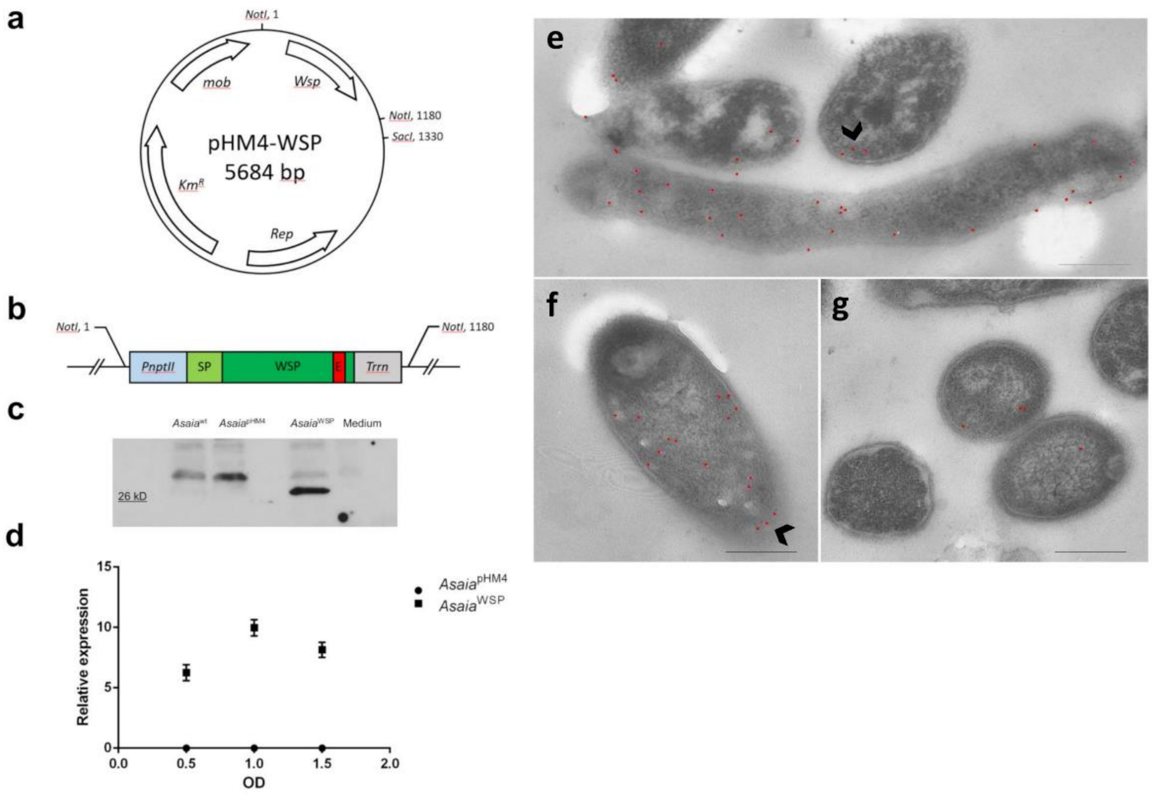

Supplementary Figure 1. WSP protein production**. a.** Map of the plasmid pHM4-WSP. A partial mob gene was present on the final construct that derived from pHM2 one [8]. WSP gene cassette orientation was assessed by PCR. **b.** Details of the WSP cassette. PnptII: neomycin phosphotranferase promoter; SP: signal peptide; WSP: *Wolbachia* surface protein; E: E-TAG epitope; Trrn: transcription terminator. E-TAG epitope was inserted in the 4th loop (L4) of the WSP sequence. Synthetic DNA were synthesized with codons harmonized with the *Asaia* sp. SF2.1 strain preferred codon usage (Accession: NZ_CBLX000000000.1 GI:737464632). **c.** Western blot analysis. The pellet of bacteria was subjected to Western blot analysis using a rabbit anti-E tag antibody. Three strains were tested: the wild strain *Asaia*^{wt}, *Asaia*^{pHM4} and *Asaia*^{WSP}. Images were taken using the same exposure time. 26 kDa represents the molecular weight of WSP protein. Bands with higher molecular weights than the WSP one (26 kDa) are related to nonspecific proteins produced by *Asaia* strains, which cross-reacted with the anti-E tag antibodies. **d.** Expression of wsp gene at different ODs. Bacteria were grown at OD 0.5, 1, 1.5 and analysed for wsp gene expression. *Asaia*^{WSP} showed a maximum expression at OD 1; no expression of wsp was detected for the *Asaia*^{pHM4}. Expression of the WSP protein by the engineered *Asaia*^{WSP} strain. **e-g.** Immunogold staining on the cultured bacteria using anti-E tag antibody. e and f panels show the staining of the *Asaia*^{WSP} (red dots indicate the gold particles

present on bacteria and arrows indicate dot groups) while panel g shows the staining of *Asaia*^{pHM4} control. Bar: 1 μm.

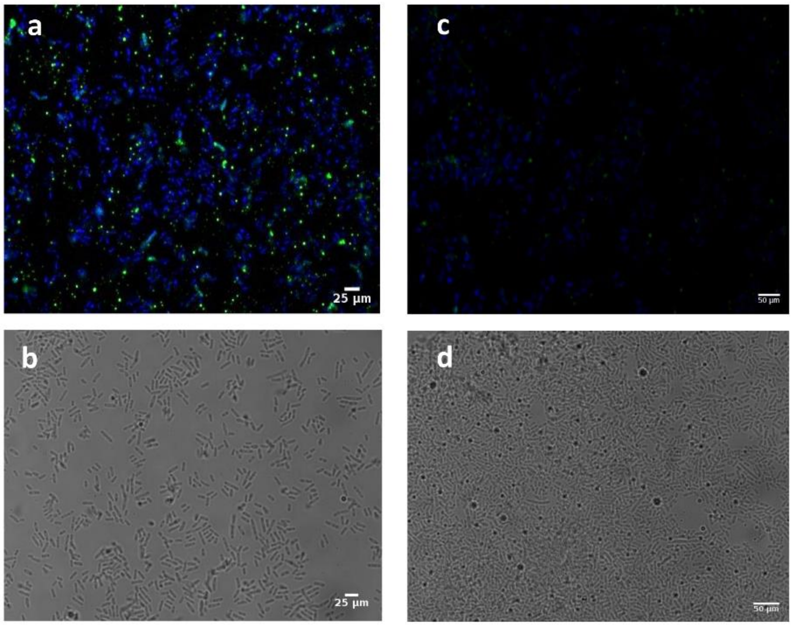

Supplementary Figure 2. Immunofluorescence assay on *Asaia*^{WSP} and *Asaia*^{pHM4} bacteria pure culture. *Asaia*^{WSP} (a, b) and *Asaia*^{pHM4} (c, d) were blocked in BSA and probed with the primary anti-E tag antibody, followed by incubation with an anti-goat IgG secondary antibody, FITC conjugate. Fluorescent images are shown on a and c panels; top and bright-field images of the same field are shown on b and d panels.

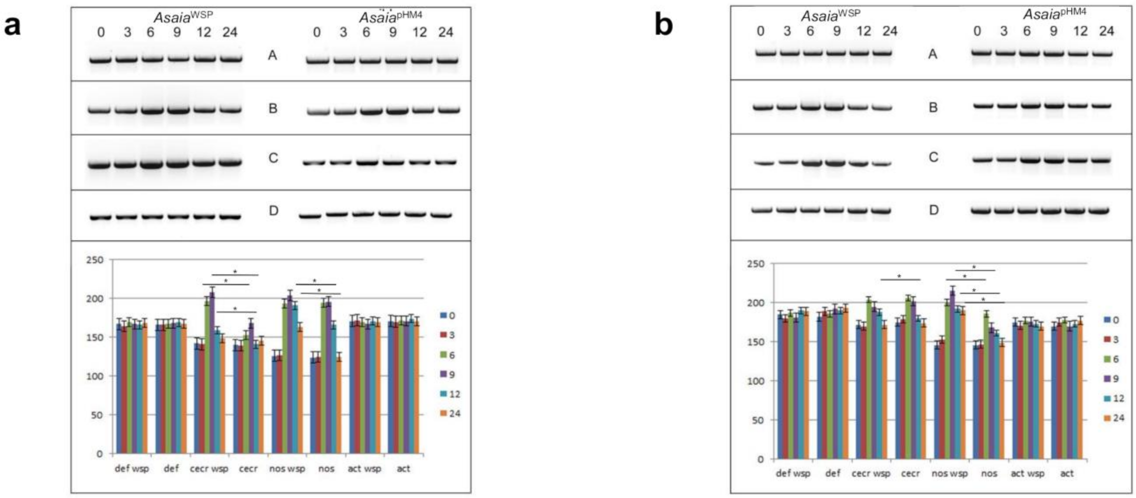

Supplementary Figure 3. Semi-quantitative analysis of the antimicrobial peptides (AMPs) defensin (A) and cecropin (B) and the nitric oxide synthase (NOS) (C) expression in hemocytes. a. Gene expression was evaluated in *Ae. aegypti* haemocytes treated with *Asaia*^{pHM4} and *Asaia*^{WSP} at different time points (0, 3, 6, 9, 12, 24 hours). Actin gene (D) was used as a constitutive control gene. b. Gene expression was evaluated in *An. stephensi* haemocytes treated with *Asaia*^{pHM4} and *Asaia*^{WSP} at different time points (0, 3, 6, 9, 12, 24 hours). Actin gene (D) was used as a constitutive control gene. The expression of immune genes was evaluated after electrophoresis in 1% agarose gel; the documentation was collected using a “Gel Doc XR” and digitally evaluated with Quantity One as schematized below each panel. Statistical analysis has been performed utilizing the analysis of variance (ANOVA) followed by Bonferroni’s multiple comparisons test ($p<0.05$ has been considered significant). One representative set of data is shown.

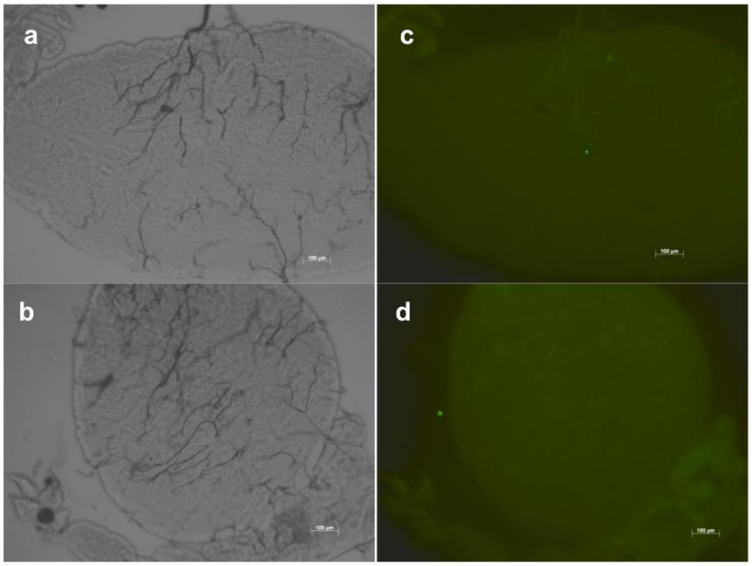

Supplementary Figure 4. Immunofluorescence controls performed on *Ae. aegypti* mosquitoes. *Ae. aegypti* female mosquitoes were fed with sugar solution for 48 hours until the midgut dissection. Dissected midguts were probed with anti-E-tag antibody, followed by incubation with a FITC-anti-goat IgG secondary antibody. Panels a and b show the bright-field of two midguts; c and d pictures display the autofluorescence of the tissues and no staining.

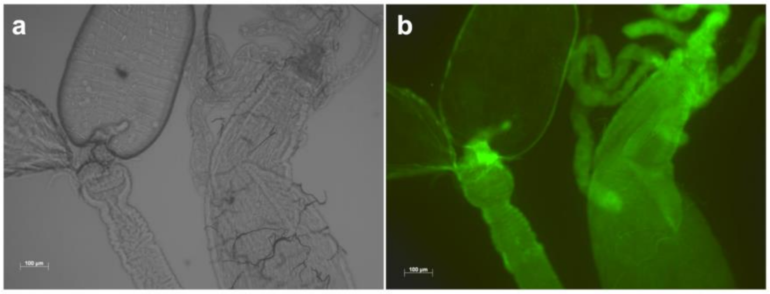

Supplementary Figure 5. Immunofluorescence controls performed on *Ae. aegypti* mosquitoes. *Ae. aegypti* female mosquitoes were fed only with a sugar solution plus kanamycin (100 µg ml^{-1}) for 48 hours until the midgut dissection. Dissected midguts were probed with anti-E-tag antibody, followed by incubation with a FITC-anti-goat IgG secondary antibody. Panels show the bright-field of a midgut; b picture displays autofluorescence of the tissues and no staining.

Supplementary Table 1. List of primers used for cloning and real-time PCR analysis here.

TARGET GENE	FORWARD SEQUENCE 5'-3'	REVERSE SEQUENCE 5'-3'	REFERENCE
wsp of Wolbachia	TCGATGATGAAGAGACCAG	GCGAGTAAAGACCTTCAATATC	This paper
16S rRNA of Asaia sp.	GCGCGTAGGCGGTTTACAC	AGCGTCAGTAATGAGCCAGGTT	[1]
rps7 of *Anopheles stephensi*	AGCAGCAGCAGCACTTGATTTG	TAAACGGCTTTCTGCGTCACCC	[2]
actin of *Anopheles stephensi*	AGCAGGAGATGGCCACC	TCCACATCTGCTGGAAGG	[2]
rps17 of Aedes aegypti	TCCGTGGTATCTCCATCAAGCT	CACTTCCGGCACGTAGTTGTC	[3]
actin of *Aedes aegypti*	GATCTGACCGACTACCTGATGA	AGATTCATCGTACTCCTGC	This paper
nitric oxide synthase of *Aedes aegypti*	TGAGTCGTTGCGCTGATTGA	TGGCAACACTGCTGTCTACC	This paper
Thioester-containing protein-1 (TEP1) of *Anopheles stephensi*	ACGACGGCTTCAATAACGAT	CCCGAGTTCCAGTTCCACTA	[4]
Anopheles Plasmodium-responsive LRR protein-1C (APL1C) of	CGTGGTAGCGTTCTCCTGAC	GCTGGGACTTCATCACAATC	[4]

Anopheles stephensi			
DEF1 of *Anopheles stephensi*	AGGCTGCGGAGAACTATC	ATAGCGACGAGCGATGCAAT	[5]
CEC1 of *Anopheles stephensi*	GCGCCCCGTTGGAAGT	TCAGGTCCGCTCCATTTATCC	[5]
NOS of *Anopheles stephensi*	GGTTCCCATCCGAAGCATT	GCAACACAGGGCAGGTTACAT	[5]
CECD *of Aedes aegypti*	ATGAACTTCACTAAGCTGTT	TCATTTTCCAATCGCTTTTAT	[6]
DEFC *of Aedes aegypti*	TTGTTTGCTTCGTTGCTCTTT	ATCTCCTACACCGAACCCACT	[6]
CLIPB37 *of Aedes aegypti*	TTGGGGGAAAACAGAAACAG	GATCTGCTTCCCAGAGAACG	[6]
NADPH oxidase (*NOXM*) of *Aedes aegypti*	TCCACAATACGGTTTCGCTA	GCCGTCCAACAGAAATTGTA	[6]
Thio-ester containing protein (TEP20) of *Aedes aegypti*	ATTTTTGACGGCTTTTGTGG	TGGATTACTTGCCCCACTTC	[7]
Transferrin of *Aedes aegypti*	AGCGAACGATGGTTTGAGTT	TATGGCATGCCTTGTACCAC	[7]

Supplementary References

1. Epis, S. *et al*. Do mosquito-associated bacteria of the genus *Asaia* circulate in humans? *Eur. J. Clin. Microbiol. Infect. Dis*. **31,** 1137-1140 (2012).
2. Capone, A. *et al*. Interactions between *Asaia*, *Plasmodium* and *Anopheles*: new insights into mosquito symbiosis and implications in malaria symbiotic control. *Parasit. Vectors* **6,** 182 (2013).
3. Fraser, J. E. *et al*. Novel *Wolbachia*-transinfected *Aedes aegypti* mosquitoes possess diverse fitness and vector competence phenotypes. *PLoS Pathog.* **13,** e1006751 (2017).
4. Zhang, J. *et al.* Modulation of *Anopheles stephensi* gene expression by nitroquine, an antimalarial drug against *Plasmodium yoelii* infection in the mosquito. *PLoS One* **9,** e89473 (2014).
5. Murdock, C. C. *et al.* Complex effects of temperature on mosquito immune function. *Proc. Biol. Sci.* **279,** 3357-3366 (2012).
6. Pan, X. *et al. Wolbachia* induces reactive oxygen species (ROS)-dependent activation of the Toll pathway to control dengue virus in the mosquito *Aedes aegypti*. *Proc. Natl. Acad. Sci. U S A* **109,** E23-E31 (2012).
7. Kambris, Z., Cook, P. E., Phuc, H. K. & Sinkins, S. P. Immune activation by life-shortening *Wolbachia* and reduced filarial competence in mosquitoes. *Science* **326,** 134-136 (2009).

8. Mostafa, H. E., Heller, K. J. & Geis, A. Cloning of *Escherichia coli* lacZ and lacY genes and their expression in *Gluconobacter* oxydans and *Acetobacter liquefaciens*. *Appl. Environ. Microbiol.* **68,** 2619-2623 (2002).
