# Supplementary material for: Chimeric symbionts expressing a Wolbachia protein stimulate mosquito immunity and inhibit filarial parasite development
Source: Commun Biol. 2020 Mar 6;3:105. doi: 10.1038/s42003-020-0835-2 (PMC7060271; doi:10.1038/s42003-020-0835-2)
Supplement: Supplementary file 2 — Description of Additional Supplementary Items [file 42003_2020_835_MOESM2_ESM.pdf]

LEGENDS FOR SUPPLEMENTARY DATA 1

Supplementary Data 1.

Source data for Figure 1: Grow rates (MGRs) of *Asaia*wt and recombinant strains (*Asaia*WSP and *Asaia*pHM4)

Source data for Figure 2: Phagocytic activity *in vitro* tests using cultured *Ae. aegypti* and *An. stephensi* haemocytes

Source data for Figure 3: qRT-PCR analyses of differentially regulated genes of *Ae. aegypti* and *An. stephensi* mosquitoes

Source data for Figure 4: *Asaia* colonization in mosquito organs

Source data for Figure 5: *Ae. aegypti* infection with transgenic bacteria and *D. immitis* microfilariae
